# Loss of glucose 6-phosphate dehydrogenase function increases oxidative stress and glutaminolysis in metastasizing melanoma cells

**DOI:** 10.1073/pnas.2120617119

**Published:** 2022-02-02

**Authors:** Arin B. Aurora, Vishal Khivansara, Ashley Leach, Jennifer G. Gill, Misty Martin-Sandoval, Chendong Yang, Stacy Y. Kasitinon, Divya Bezwada, Alpaslan Tasdogan, Wen Gu, Thomas P. Mathews, Zhiyu Zhao, Ralph J. DeBerardinis, Sean J. Morrison

**Affiliations:** ^a^Children’s Research Institute and Department of Pediatrics, University of Texas Southwestern Medical Center, Dallas, TX 75390;; ^b^Department of Dermatology, University of Texas Southwestern Medical Center, Dallas, TX 75390;; ^c^Eugene McDermott Center for Human Growth and Development, University of Texas Southwestern Medical Center, Dallas, TX 75390;; ^d^Howard Hughes Medical Institute, University of Texas Southwestern Medical Center, Dallas, TX 75390

**Keywords:** melanoma, metastasis, oxidative stress, pentose phosphate pathway, glutaminolysis

## Abstract

The pentose phosphate pathway is a major source of NADPH for oxidative stress resistance in cancer cells but there is limited insight into its role in metastasis, when some cancer cells experience high levels of oxidative stress. To address this, we mutated the substrate binding site of glucose 6-phosphate dehydrogenase (G6PD), which catalyzes the first step of the pentose phosphate pathway, in patient-derived melanomas. *G6PD* mutant melanomas had significantly decreased G6PD enzymatic activity and depletion of intermediates in the oxidative pentose phosphate pathway. Reduced G6PD function had little effect on the formation of primary subcutaneous tumors, but when these tumors spontaneously metastasized, the frequency of circulating melanoma cells in the blood and metastatic disease burden were significantly reduced. *G6PD* mutant melanomas exhibited increased levels of reactive oxygen species, decreased NADPH levels, and depleted glutathione as compared to control melanomas. *G6PD* mutant melanomas compensated for this increase in oxidative stress by increasing malic enzyme activity and glutamine consumption. This generated a new metabolic vulnerability as *G6PD* mutant melanomas were more dependent upon glutaminase than control melanomas, both for oxidative stress management and anaplerosis. The oxidative pentose phosphate pathway, malic enzyme, and glutaminolysis thus confer layered protection against oxidative stress during metastasis.

## Significance

Melanoma metastasis is limited by oxidative stress. Cells that enter the blood experience high levels of reactive oxygen species and usually die of ferroptosis. We found that melanoma cells become more dependent upon the oxidative pentose phosphate pathway to manage oxidative stress during metastasis. When pentose phosphate pathway function was impaired by reduced glucose 6-phosphate dehydrogenase (*G6PD*) function, melanoma cells increased malic enzyme activity and glutamine consumption. Melanoma cells thus have redundant and layered protection against oxidative stress.

The pentose phosphate pathway is an important source of NADPH for oxidative stress resistance (1–5). The oxidative branch of the pentose phosphate pathway contains two enzymes that generate NADPH from NADP^+^, glucose 6-phosphate dehydrogenase (G6PD) and 6-phosphogluconate dehydrogenase (PGD) (*SI Appendix*, Fig. S1). NADPH is an important source of reducing equivalents for oxidative stress resistance because it is used by cells to convert oxidized glutathione (GSSG) to glutathione (GSH), an abundant redox buffer. Complete deficiency for G6PD is embryonic-lethal in mice (2, 6, 7) but hypomorphic *G6PD* mutations are common in certain human populations, perhaps because they protect against malaria (8, 9). These partial loss-of-function *G6PD* mutations are well tolerated in adults, though they sensitize red blood cells to hemolysis from oxidative stress under certain circumstances (10).

Several studies have reported a lower incidence and mortality for certain cancers in people with hypomorphic mutations in *G6PD* (11–14), suggesting that cancer cells depend upon G6PD to manage oxidative stress. Cells experience high levels of oxidative stress during certain phases of cancer development and progression, including during metastasis (15–17). Antioxidant mechanisms thus promote the survival of cells during oncogenic transformation (18, 19) as well as during metastasis (15, 16). For example, relative to primary cutaneous tumors, metastasizing melanoma cells exhibit increased dependence upon the folate pathway (15), monocarboxylate transporter-1 (MCT1) (20), and glutathione peroxidase-4 (GPX4) (21), each of which directly or indirectly attenuate oxidative stress. By better understanding the mechanisms that confer oxidative stress resistance in cancer cells, it may be possible to develop pro-oxidant therapies that inhibit cancer progression by exacerbating the oxidative stress experienced by cancer cells.

G6PD (22) or PGD deficiency (23–25) reduce the growth of some cancers, including melanoma, but G6PD deficiency has little effect on primary tumor formation by K-Ras–driven epithelial cancers (26). This is at least partly because loss of G6PD in these cancers leads to compensatory increases in the function of other NADPH-generating enzymes, including malic enzyme and isocitrate dehydrogenase (1, 27). Nonetheless, pentose phosphate pathway function may increase during metastasis (20, 28–30) and higher G6PD expression is associated with worse outcomes in several cancers (31–33), raising the question of whether metastasizing cells are particularly dependent upon G6PD. G6PD is not essential for metastasis in a breast cancer cell line but it reduces their capacity to form metastatic tumors (26).

Melanoma cells show little evidence of oxidative stress in established primary tumors but exhibit increased levels of reactive oxygen species (ROS) and dependence upon antioxidant mechanisms during metastasis (15, 20, 21). To test if these cells are more dependent upon the pentose phosphate pathway during metastasis, we generated three *G6PD* mutant melanomas, including two patient-derived xenografts and one human melanoma cell line. Reduced G6PD function had little effect on the formation or growth of primary subcutaneous tumors but significantly increased ROS levels and reduced spontaneous metastasis. *G6PD* mutant melanomas compensated by increasing malic enzyme activity and glutamine consumption, both to increase oxidative stress resistance and to replenish tricarboxylic acid (TCA) cycle intermediates through anaplerosis. Melanoma cells thus have redundant layers of protection against oxidative stress during metastasis, including the abilities to alter fuel consumption and antioxidant pathway utilization.

## Results

### Pentose Phosphate Pathway Metabolites Are Enriched in Metastases.

**Fig. 1.**
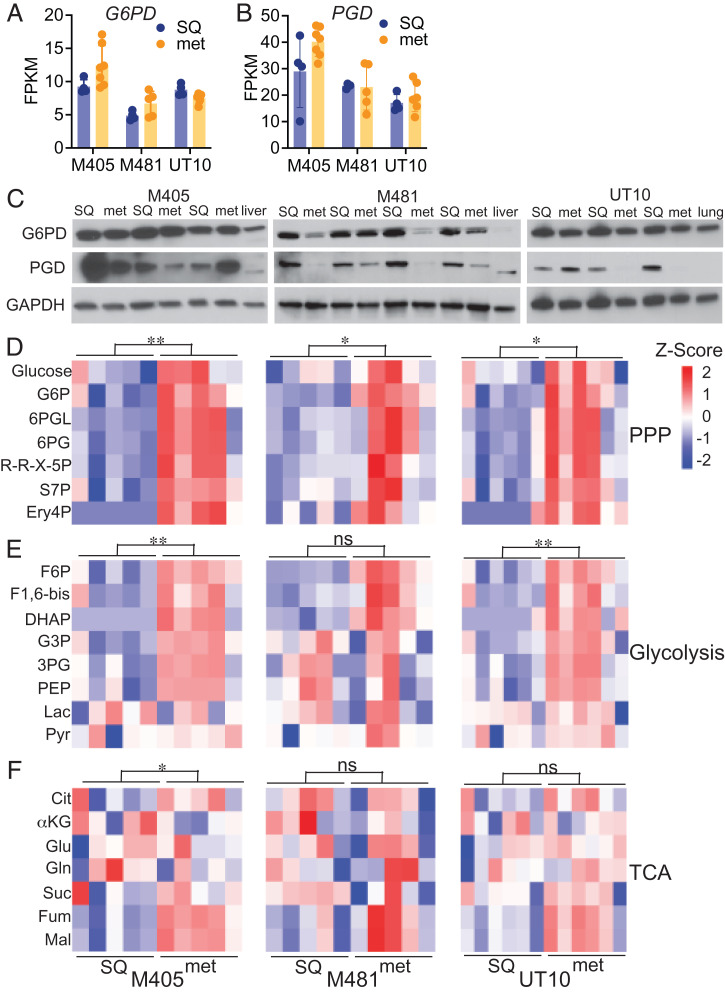
Pentose phosphate pathway metabolites are more abundant in metastatic as compared to subcutaneous primary melanomas. (*A–C*) Subcutaneous tumors (SQ) and metastatic nodules (met) were harvested from the same mice xenografted subcutaneously with M405, M481, and UT10 patient-derived melanomas. *G6PD* (*A*) and *PGD* (*B*) transcripts were assessed by RNA-sequencing analysis (*n* = 4 to 7 samples per melanoma). (*C*) Western blot analysis of G6PD and PGD in subcutaneous and metastatic tumors from mice xenografted with M405, M481, and UT10 melanomas. Each pair of adjacent subcutaneous and metastatic samples were obtained from the same mouse (*n* = 3 to 4 mice per melanoma). GAPDH is shown as a loading control. Lysates from normal mouse liver or lung are included as controls. (*D–F*) Relative levels of pentose phosphate pathway (PPP) (*D*), glycolytic (*E*), and TCA cycle (*F*) metabolites in subcutaneous and metastatic tumor specimens obtained from mice xenografted with M405 (*Left*), M481 (*Center*), or UT10 (*Right*) melanomas. Each column represents a different tumor from a different mouse. Metabolite abbreviations are defined in *SI Appendix*, Fig. S1. The statistical significance of each pathway was assessed by comparing the major effect between subcutaneous and metastatic tumors using a repeated-measure two-way ANOVA. Data represent mean ± SD. Statistical significance was assessed using Student's *t* tests (*A* and *B*) or a two-way repeated-measures ANOVA on the log_2_-transformed data (*D–F*). Statistical tests were two-sided. Multiple comparisons were adjusted by the FDR method. *P < 0.05, **P < 0.01, ns = not significant.

To compare the levels of pentose phosphate pathway metabolites (*SI Appendix*, Fig. S1) in subcutaneous tumors and metastatic nodules, we subcutaneously transplanted efficiently metastasizing melanomas obtained from three patients (M405, M481, and UT10) into NOD/SCID IL2Rγ^null^ (NSG) mice, allowing them to form primary subcutaneous tumors and to spontaneously metastasize. All of the melanomas were tagged with constitutive DsRed and luciferase expression, unambiguously distinguishing these cells from recipient mouse cells (see *SI Appendix*, Fig. S2 *A* and *B* for flow cytometry gating strategies) and enabling the quantitation of metastatic disease burden by bioluminescence imaging (*SI Appendix*, Fig. S2 *C* and *D*). We dissected subcutaneous tumors and small metastatic nodules (<3 mm in diameter) from the same mice and compared G6PD and PGD expression. G6PD and PGD were consistently expressed by both primary subcutaneous tumors and metastatic nodules at the RNA (Fig. 1 *A* and *B*) and protein levels (Fig. 1*C*). Expression levels varied among tumors but did not differ in any consistent way between subcutaneous tumors and metastatic nodules.

We performed a targeted metabolomic analysis on primary subcutaneous tumors and metastatic nodules and found that levels of pentose phosphate pathway metabolites were significantly higher in metastatic nodules as compared to subcutaneous tumors in all three melanomas (Fig. 1*D*). The levels of glycolytic intermediates were also significantly higher in metastatic nodules as compared to subcutaneous tumors in two of the melanomas (Fig. 1*E*). TCA cycle intermediates were elevated in metastatic nodules from one melanoma (Fig. 1*F*). The consistently higher levels of pentose phosphate pathway metabolites in most metastatic nodules raised the possibility that the pathway was more active in metastatic as compared to primary subcutaneous tumors.

### G6PD Mutant Melanomas.

**Fig. 2.**
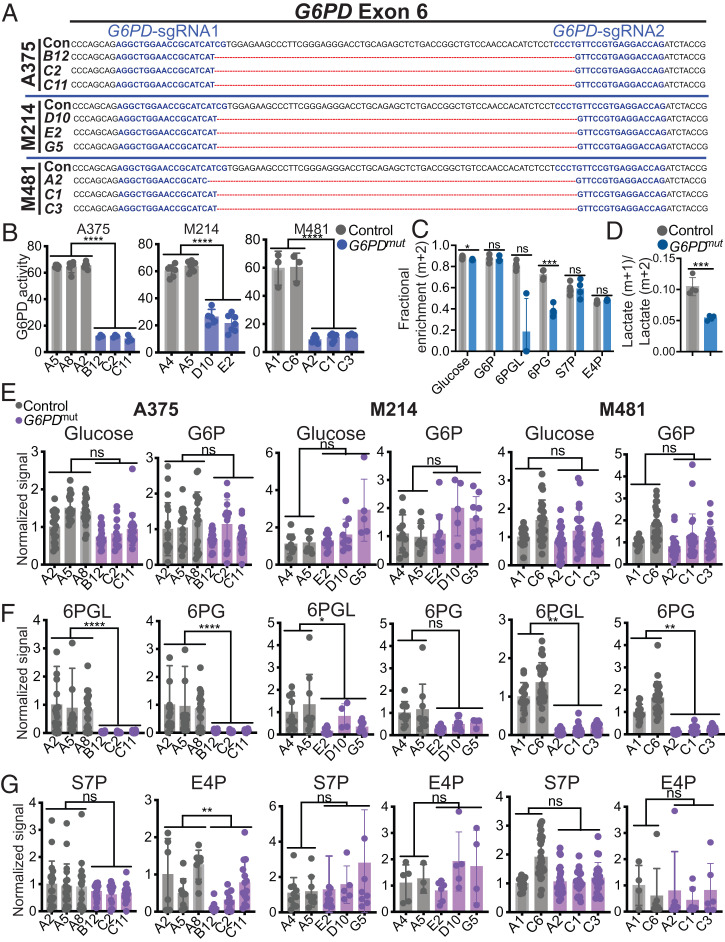
Generation of *G6PD* mutant melanoma cells with impaired oxidative pentose phosphate pathway function. (*A*) CRISPR editing of *G6PD* in three melanomas (A375, M214, and M481) to generate three independently targeted clones per melanoma with 66- to 68-bp deletions in exon 6. A375 was derived from a female patient while M214 and M481 are from males. Guide sequences are highlighted in blue. The 66- to 68-bp region deleted in the mutants is depicted by red lines. The control (Con) sequence is shown for reference. (*B*) G6PD enzymatic activity in subcutaneous tumors formed by *G6PD* mutant or control melanoma cells. (*C* and *D*) Tracing of [1,2-^13^C] glucose in *G6PD* mutant or control A375 melanoma cells to assess the activity of the pentose phosphate pathway. The data show the fractional enrichments (m+2) in glucose, G6P, 6PGL, 6PG, E4P, S7P (*C*) and the ratio of m+1 lactate to m+2 lactate (*D*) 4 h after adding labeled glucose to culture. (*E–G*) Analysis of the relative levels of glucose, G6P, 6PGL, 6PG, and S7P, and E4P levels in subcutaneous tumors formed by *G6PD* mutant and control A375 (*Left*), M214 (*Center*), and M481 (*Right*) melanoma cells (*n* = 5 to 8 tumors per clone). Each dot represents a different tumor from a different mouse. Data represent mean ± SD. Statistical significance was assessed using linear mixed-effects analysis (*B*), linear mixed-effects analysis on log_2_-transformed data (*E–G*), Student’s *t* tests (*D*), or Mann–Whitney *U* tests (*C*). Statistical tests were two-sided. Multiple comparisons were adjusted by the FDR method. *P < 0.05, **P < 0.01, ***P < 0.001, ****P < 0.0001, ns = not significant.

To test whether melanoma metastasis depended on the oxidative pentose phosphate pathway, we used CRISPR to make small deletions in exon 6 of *G6PD* in three melanomas including the A375 human melanoma cell line and two patient-derived melanomas (M481 and M214). Exon 6 encodes the substrate binding domain and mutations in exon 6 severely reduce G6PD enzymatic activity (34). We obtained three independently targeted clones for each melanoma and confirmed by sequencing that they had 66- to 68-bp deletions in exon 6 (Fig. 2*A*). The *G6PD* mutant clones had greatly reduced levels of G6PD enzymatic activity (Fig. 2*B*).

To assess the effect of the *G6PD* mutations on pentose phosphate pathway function, we performed isotope tracing by supplementing A375 cells with [1,2-^13^C] glucose in culture, then compared the fractional enrichment in pentose phosphate pathway metabolites in *G6PD* mutant as compared to control clones. We observed little or no difference between *G6PD* mutant and control cells in the fractional enrichments (m+2) of glucose, glucose-6-phosphate, or in the nonoxidative pentose phosphate pathway intermediates sedoheptulose 7-phosphate (S7P) or erythrose 4-phosphate (E4P); however, the fractional enrichments in the oxidative pentose phosphate pathway intermediates 6-phosphogluconolactone (6PGL) and 6-phosphogluconate (6PG) were lower in the *G6PD* mutant as compared to control clones (Fig. 2*C*). We also compared the relative flux of labeled glucose through glycolysis versus the oxidative pentose phosphate pathway by comparing the ratio of M+1 lactate (derived from the oxidative pentose phosphate pathway) to M+2 lactate (derived from glycolysis) (35). This ratio was significantly reduced in *G6PD* mutant as compared to control cells (Fig. 2*D*). Reduced G6PD function thus impaired the function of the oxidative branch of the pentose phosphate pathway, as expected.

We performed metabolomic analysis on primary subcutaneous tumors formed by the *G6PD* mutant as compared to control melanoma cells. The levels of glucose and glucose-6-phosphate (Fig. 2*E*) and the nonoxidative pentose phosphate pathway intermediates S7P and E4P (Fig. 2*G*) generally did not significantly differ between the *G6PD* mutant and control tumors. Consistent with the isotope tracing data, metabolites within the oxidative pentose phosphate pathway, 6PGL and 6PG, were almost always significantly depleted within the *G6PD* mutant as compared to control tumors (Fig. 2*F*). Thus, both isotope tracing and metabolomics data suggest that *G6PD* mutant melanomas have diminished flux through the oxidative pentose phosphate pathway.

### Metastasizing Melanoma Cells Depend on G6PD.

**Fig. 3.**
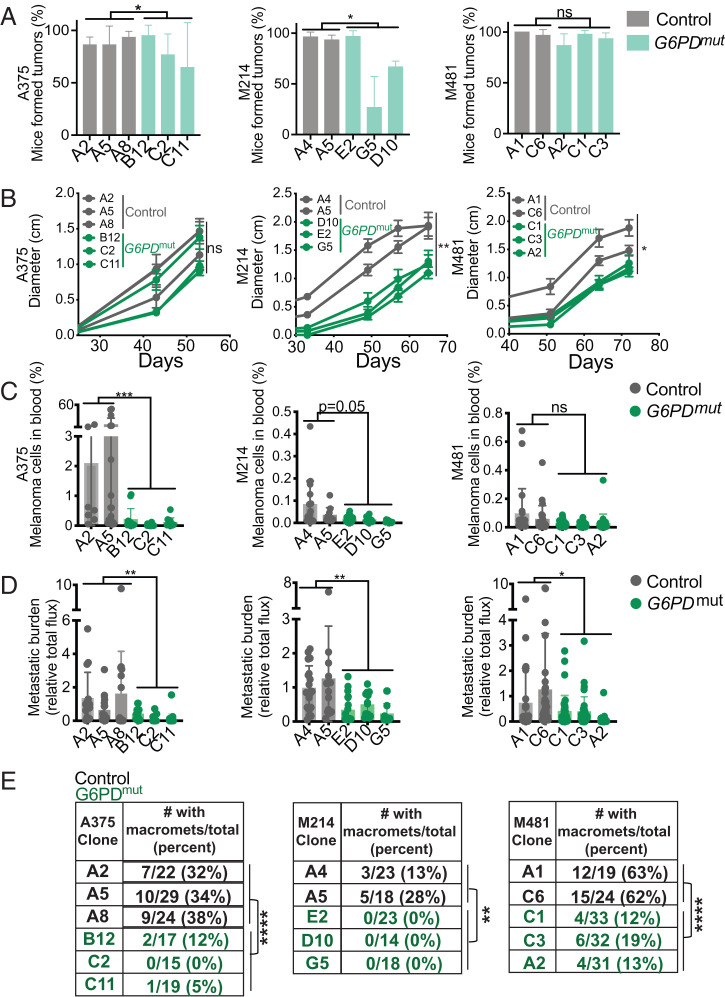
*G6PD* deficiency has limited effects on the growth of subcutaneous tumors but impairs metastasis. (*A–E*) NSG mice were injected subcutaneously (100 cells per injection) with *G6PD* mutant or control cells from A375, M214, and M481 melanomas and the tumors were allowed to spontaneously metastasize. (*A*) The percentage of mice that formed subcutaneous tumors. (*B*) The growth of subcutaneous tumors. (*C*) The frequency of circulating melanoma cells in the blood. (*D*) Metastatic disease burden by bioluminescence imaging. (*E*) The percentage of mice that formed macrometastases. Three independent experiments were performed per melanoma, but *B* reflects results from a single representative experiment per melanoma due to the difficulty of combining tumor growth rate data from independent experiments. Data represent mean ± SD. Statistical significance was assessed using generalized linear mixed-effects analysis (*A* and *E*), linear mixed-effects analysis (*B*), or linear mixed-effects analysis on log_2_-transformed data (*C* and *D*). Statistical tests were two-sided. Multiple comparisons were adjusted by the FDR method. *P < 0.05, **P < 0.01, ***P < 0.001, ****P < 0.0001, ns = not significant.

To test whether G6PD deficiency affected subcutaneous tumor growth or metastasis, we subcutaneously transplanted 100 *G6PD* mutant or control melanoma cells into NSG mice. Nearly all of the injections of control cells and most of the injections of *G6PD* mutant cells formed tumors, though some clones of *G6PD* mutant A375 and M214 cells formed fewer tumors (Fig. 3*A*). The *G6PD* mutant and control A375 tumors grew at similar rates, though the *G6PD* mutant M214 and M481 tumors grew more slowly than control tumors (Fig. 3*B*). Once tumors reached 2.5 cm, we assessed spontaneous metastasis by measuring the frequency of circulating melanoma cells in the blood and the metastatic disease burden using bioluminescence imaging of organs. The frequency of circulating melanoma cells in the blood was generally lower in mice with *G6PD* mutant as compared to control melanomas (see Fig. 3*C* and *SI Appendix*, Fig. S2*B* for flow cytometry gating strategy). Metastatic disease burden (Fig. 3*D*) and the percentage of mice that spontaneously formed macrometastases (Fig. 3*E*) was always significantly lower in mice with the *G6PD* mutant as compared to control melanomas. In the case of A375, it is particularly striking that subcutaneous tumors formed by *G6PD* mutant cells grew at a similar rate as tumors formed by control cells, and yet the frequency of circulating melanoma cells in the blood, overall metastatic disease burden, and the percentage of mice that formed macrometastases were all significantly lower in mice with the *G6PD* mutant as compared to control cells. The data suggest that melanoma cells become more dependent upon G6PD during metastasis, consistent with the increase in oxidative stress during metastasis (15, 20, 21).

**Fig. 4.**
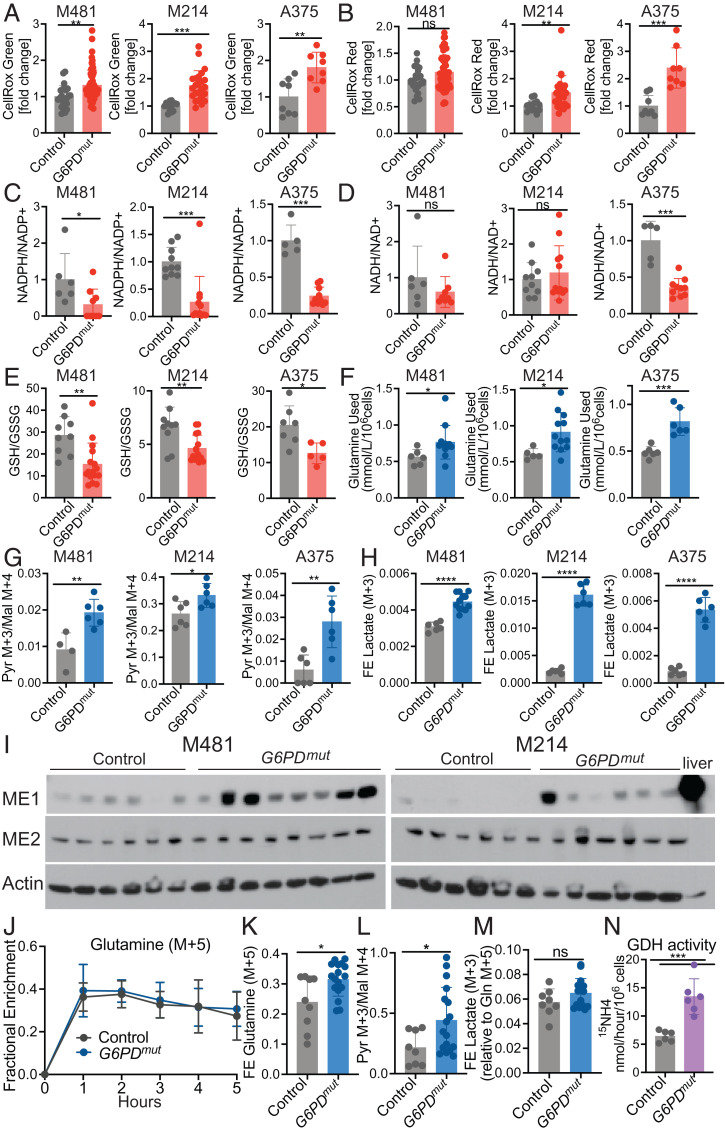
Reduced *G6PD* function induces oxidative stress and increases glutamine utilization. (*A* and *B*) ROS levels based on median fluorescence intensity of CellRox green (*A*) or CellRox red (*B*) in subcutaneous tumors formed by *G6PD* mutant or control melanoma cells (*n* = 2 to 3 clones per genotype per melanoma with five mice per clone in two to three independent experiments per melanoma). Each dot represents data from one mouse. (*C–E*) The relative ratios of NADPH to NADP^+^ (*C*), NADH to NAD^+^ (*D*), and the absolute molar ratios of GSH to GSSG quantified with an internal standard (*E*) in *G6PD* mutant or control M481, M214, and A375 melanoma cells (three independent experiments were performed per melanoma and one representative experiment per melanoma is shown; *n* = 5 to 15 mice per group). (*F*) The amount of glutamine consumed from the culture medium is shown as mmol/L/10^6^ cells for *G6PD* mutant and control melanomas after 24 (M481 and M214) or 8 h (A375) in culture (*n* = 2 clones per genotype per melanoma, with three to six replicate cultures per clone in two independent experiments per melanoma). (*G* and *H*) *G6PD* mutant or control melanoma cells were cultured with [U-^13^C] glutamine and the ratio of pyruvate (m+3) to malate (m+4) (*G*) and fractional enrichment in lactate (m+3) were measured (*H*) (*n* = 2 clones per genotype per melanoma, with six replicate cultures per clone; one representative experiment is shown from two to three independent experiments per melanoma). (*I*) Western blot analysis of ME1 and ME2 in lysates of subcutaneous tumors. Actin is a loading control. Each lane is a different tumor from a different mouse. Normal mouse liver is shown as a positive control. (*J–M*) NSG mice xenografted with *G6PD* mutant or control M481 melanoma cells were infused with [U-^13^C] glutamine. The fractional enrichment of glutamine m+5 in the blood (*J*) and in subcutaneous tumors after 5 h of infusion (*K*). The ratio of pyruvate (m+3) to malate (m+4) (*L*) and fractional enrichment of lactate (m+3) normalized to the fractional enrichment of glutamine (m+5) (*M*) (*n* = 2 clones per genotype per melanoma, with four to eight mice per clone in two independent experiments). Each dot represents a different tumor in a different mouse. (*N*) *G6PD* mutant and control melanoma cells were cultured in medium supplemented with L-[α-^15^N]glutamine and GDH activity was measured based on the rate at which ^15^N was transferred from glutamine to ^15^NH_4_ (two clones per genotype from three independent experiments; each dot represents cells from a different culture; *n* = 3 wells per clone). Data represent mean ± SD. Statistical significance was assessed using Mann–Whitney *U* tests (*A–C*, *J*, and *M*), Student’s *t* tests on log_2_-transformed data (*D*–*F*), Student’s *t* tests (*G*, *H*, and *K–M*), Welch's *t* test (*H*), or Student’s *t* tests on log_2_-transformed data (*N*). Statistical tests were two-sided. Multiple comparisons were adjusted by the FDR method: *P < 0.05, **P < 0.01, ***P < 0.001, ****P < 0.0001, ns = not significant.

Levels of ROS tended to be significantly higher in subcutaneous tumors formed by the *G6PD* mutant as compared to control melanoma cells based on both CellRox green (Fig. 4*A*) and CellRox red (Fig. 4*B*) staining. The *G6PD* mutant melanomas consistently had lower NADPH/NADP^+^ ratios as compared to control cells (Fig. 4*C*), primarily due to a decrease in NADPH levels in the *G6PD* mutant melanomas. NADH/NAD^+^ ratios did not significantly differ except in A375 cells (Fig. 4*D*). The ratios of GSH to GSSG were significantly lower in *G6PD* mutant as compared to control melanomas (Fig. 4*E*). The data thus suggest that melanoma cells experienced higher levels of oxidative stress when G6PD function was decreased.

### G6PD Mutant Melanomas Increase Glutaminolysis.

We wondered whether *G6PD* mutant melanomas might compensate for the loss of G6PD function by increasing NADPH production through other mechanisms. *G6PD*-deficient colon and lung cancer cells increase malic enzyme and isocitrate dehydrogenase activity (1, 27). We first compared the consumption of glutamine by *G6PD* mutant and control melanoma cells in culture. We found that the *G6PD* mutant cells consistently consumed more glutamine from the culture medium than control cells (Fig. 4*F*).

To test if *G6PD* mutant melanomas compensate by increasing glutaminolysis (the conversion of glutamine to pyruvate through the TCA cycle, with the production of NADPH by malic enzyme) (*SI Appendix*, Fig. S1), we performed isotope tracing with [U-^13^C] glutamine in culture. [U-^13^C] glutamine gives rise to 4-carbon TCA cycle intermediates, such as malate (m+4). Because malic enzyme decarboxylates malate to produce pyruvate, malic enzyme activity is reflected in the ratio of pyruvate m+3 to malate m+4. This ratio was consistently and significantly higher in *G6PD* mutant as compared to control melanoma cells (Fig. 4*G*). The fractional enrichment of (m+3) lactate was also significantly higher in the *G6PD* mutant as compared to control melanoma cells (Fig. 4*H*). Malic enzyme 2 (ME2), which localizes to mitochondria, was expressed by all melanomas at similar levels (Fig. 4*I*). ME1, which localizes to the cytoplasm, was expressed by most melanomas but it tended to higher in *G6PD* mutant as compared to control melanomas (Fig. 4*I*). These data suggest that *G6PD* mutant melanomas compensate for the loss of G6PD function by increasing glutaminolysis and malic enzyme activity, partly by increasing ME1 levels.

To assess this in vivo, we infused [U-^13^C] glutamine into mice xenografted with *G6PD* mutant or control M481 melanomas (36). Infusion of [U-^13^C] glutamine enriched the circulating glutamine (Fig. 4*J*), lactate (*SI Appendix*, Fig. S3*A*), and pyruvate (*SI Appendix*, Fig. S3*C*) pools in the blood to a similar extent in mice transplanted with *G6PD* mutant and control melanomas. Consistent with the results in culture, the fractional enrichment of glutamine (m+5) in tumors was significantly higher in the *G6PD* mutant as compared to control melanomas (Fig. 4*K*). Malic enzyme activity, based on the ratio of pyruvate (m+3) to malate (m+4), was significantly higher in the *G6PD* mutant as compared to control melanoma cells (Fig. 4*L*). This ratio was higher in vivo than in the same melanomas cultured with glutamine (m+5) in vitro (compare Fig. 4*L* and Fig. 4 *G*, *Left*), regardless of *G6PD* status. This difference likely results in part from glutaminolysis and gluconeogenesis occurring outside the tumor. These pathways can produce circulating glucose (m+3), lactate (m+3), and pyruvate (m+3) from [U-^13^C] glutamine that enters the tumor (37). Nevertheless, while the elevated pyruvate (m+3) to malate (m+4) ratio in *G6PD* mutant as compared to control melanomas may have been influenced by circulating lactate/pyruvate (m+3), the data suggest a relative increase in malic enzyme’s contribution to the pyruvate pool in the *G6PD* mutant tumors because the levels of lactate (m+3) and pyruvate (m+3) in the blood did not differ between mice with *G6PD* mutant and control melanomas. The fractional enrichment of lactate (m+3) was also higher in *G6PD* mutant as compared to control tumors (*SI Appendix*, Fig. S3*B*), though the difference was not statistically significant when normalized to glutamine fractional enrichment (Fig. 4*M*).

Cells convert glutamine into the TCA cycle intermediate α-ketoglutarate (αKG) via glutaminase (GLS) and glutamate dehydrogenase (GDH) (*SI Appendix*, Fig. S1). GDH converts glutamate to αKG while generating ammonia (NH_3_) from the α-nitrogen of glutamine. To test if *G6PD* mutant melanomas exhibit higher GDH activity, we cultured cells with ^15^*N*-glutamine (labeled in the α-position) and measured the production of ^15^NH_4_^+^ by gas chromatography/mass spectrometry (GC/MS) (38). GDH activity was significantly increased in the *G6PD* mutant as compared to control melanomas (Fig. 4*N*). This suggests that *G6PD* mutant melanomas increase both GDH and malic enzyme activity.

We assessed the contribution of glutamine (m+5) to TCA cycle intermediates in *G6PD* mutant and control melanomas growing subcutaneously. Whether we examined fractional enrichments normalized to glutamine (m+5) levels in the tumor cells (*SI Appendix*, Fig. S3 *E* and *F*) or nonnormalized fractional enrichments (*SI Appendix*, Fig. S3 *G* and *H*), we did not observe significant differences in the contribution of labeled glutamine to TCA cycle intermediates in the *G6PD* mutant as compared to control melanomas. Nonetheless, the nonnormalized fractional enrichments tended to be higher in the *G6PD* mutant as compared to control melanomas (*SI Appendix*, Fig. S3 *G* and *H*).

### G6PD Deficiency Sensitizes Melanoma Cells to Glutaminase Inhibition.

**Fig. 5.**
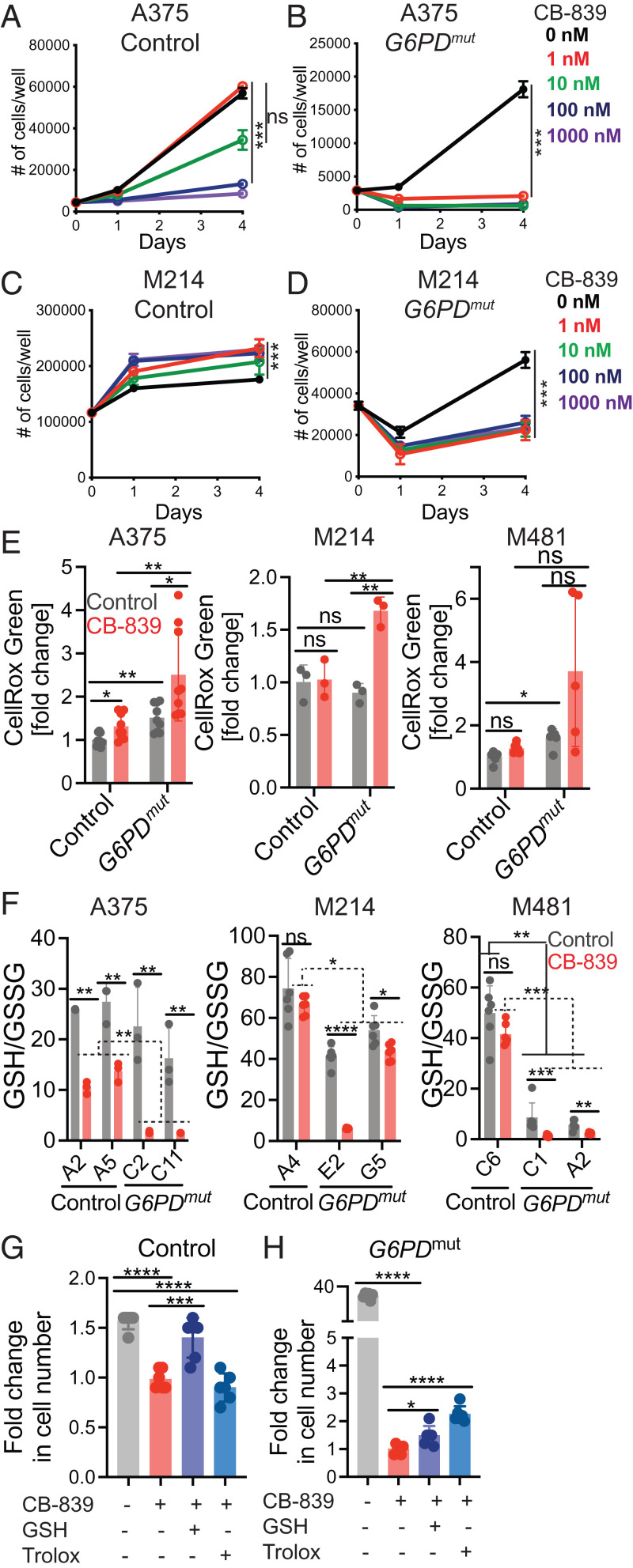
*G6PD* deficiency sensitizes melanoma cells to glutaminase inhibition. (*A–D*) *G6PD* mutant and control A375 (*A* and *B*) or M214 (*C* and *D*) melanoma cells were cultured with the glutaminase inhibitor, CB-839 (1 nM, 10 nM, 100 nM, or 1,000 nM), or vehicle (DMSO) control (0 nM for CB-839) and the number of cells in each well was counted over time. (*E* and *F*) A375, M214, or M481 melanoma cells were cultured in the presence of CB-839 (100 nM) or vehicle for 24 h and ROS levels were analyzed based on flow cytometric analysis of CellRox green staining (*E*) and the absolute molar ratios of GSH to GSSG were quantified by MS with an internal standard (*F*). (*G* and *H*) A375 control (*G*) and *G6PD* mutant (*H*) melanoma cells were cultured with the glutaminase inhibitor CB-839 (1 nM) or vehicle (DMSO) control with or without glutathione ethyl ester (GSH, 500 μM) or Trolox (25 μM) for 4 d. Data are shown as the fold-change in cell number relative to CB-839 treatment alone. Each panel reflects data from a single experiment that is representative of two or three independent experiments, with one to two clones per genotype and three to six replicate cultures per clone per experiment. All data represent mean ± SD. Statistical significance was assessed using nparLD (*A–D*), Welch's one-way ANOVA with log_2_-transformed data followed by Dunnett’s T3 test for multiple comparisons adjustment (*E*), Welch’s *t* tests with log_2_-transformed data (*F*), or one-way ANOVA followed by Dunnett’s T3 test for multiple comparisons adjustment (*G* and *H*). Statistical tests were two-sided. Multiple comparisons were adjusted by the FDR method except for *E, G,* and *H*: *P < 0.05, **P < 0.01, ***P < 0.001, ****P < 0.0001, ns = not significant.

Glutamine dependency has been targeted in several cancers, primarily by inhibiting glutaminase (39). To test whether *G6PD* mutant melanoma cells were sensitive to glutaminase inhibition, we cultured *G6PD* mutant and control melanoma cells in vehicle (DMSO) or CB-839, a glutaminase inhibitor (40); 1 nM CB-839 did not significantly affect the growth of A375 control melanoma cells and 10 nM CB-839 only modestly reduced their growth (Fig. 5*A*). In contrast, 1 nM and 10 nM CB-839 completely blocked the growth of *G6PD^mut^* A375 cells (Fig. 5*B*). No concentration of CB-839 reduced the growth of M214 control cells (Fig. 5*C*); however, all concentrations of CB-839 (1 to 1,000 nM) significantly reduced the growth of *G6PD* mutant M214 cells (Fig. 5*D*). *G6PD* mutant melanoma cells were therefore far more sensitive to glutaminase inhibition than control melanoma cells.

To test if glutaminase inhibition increased oxidative stress in *G6PD* mutant melanoma cells, we measured ROS levels (CellRox green) and GSH to GSSG ratios in *G6PD* mutant and control melanoma cells treated in culture with 100 nM CB-839 or vehicle control. CB-839 modestly increased ROS levels in A375 control cells but not in M214 or M481 control cells (Fig. 5*E*). ROS levels were usually higher in the *G6PD* mutant as compared to control melanoma cells (including A375 and M481, but not M214) (Fig. 5*E*). ROS levels were highest in *G6PD* mutant A375, M214, and M481 cells treated with CB-839, generally higher than in untreated *G6PD* mutant cells or in CB-839 treated control cells (Fig. 5*E*). Glutaminase inhibition thus increased ROS levels to a greater extent in *G6PD* mutant as compared to control melanoma cells.

CB-839 treatment significantly reduced GSH/GSSG ratios in A375 control cells but not in M214 or M481 control cells (Fig. 5*F*). *G6PD* deficiency reduced GSH/GSSG ratios in M481 cells but not in A375 or M214 cells (Fig. 5*F*). GSH/GSSG ratios were substantially lower in *G6PD* mutant A375, M214, and M481 cells treated with CB-839, as compared to either untreated *G6PD* mutant cells or CB-839 treated control cells (Fig. 5*F*). Glutaminase inhibition typically reduced GSH/GSSG ratios to a greater extent in the *G6PD* mutant as compared to control melanoma cells. Taken together, the data suggest that reduced G6PD function renders melanoma cells more dependent upon glutaminolysis.

To test if antioxidants would rescue the effect of glutaminase inhibition on the growth of *G6PD* mutant and control melanomas, we treated A375 cells with a low concentration of CB-839 (1 nM). Addition of glutathione ethyl ester, a cell-permeable form of GSH, but not Trolox, rescued the effect of glutaminase inhibition on the growth of control cells (Fig. 5*G*). Both GSH and Trolox partially rescued the much more profound effects of glutaminase inhibitor on the growth of *G6PD* mutant melanoma cells (Fig. 5*H*). Oxidative stress thus appears to have contributed to the effects of glutaminase inhibition on the growth of *G6PD* mutant and control melanoma cells. However, the magnitude of the rescue in *G6PD* mutant cells was small, raising the possibility that glutamine consumption promoted the growth of *G6PD* mutant melanoma cells through additional mechanisms beyond its role in suppressing oxidative stress.

A recent study (27) observed that in cancers with NRF2 activation, loss of G6PD activity depleted TCA cycle intermediates as a result of increased malic enzyme and isocitrate dehydrogenase activity and increased the dependence on glutamine as a source of TCA cycle intermediates. To test whether this contributed to the dependence of *G6PD* mutant melanomas upon glutaminolysis, we compared the abundance of TCA cycle intermediates between *G6PD* mutant and control melanomas growing subcutaneously. In A375 tumors, *G6PD* mutant cells had significantly lower levels of glutamate, succinate, fumarate, malate, and citrate (*SI Appendix*, Fig. S4*A*); however, in M214 and M481 melanomas, *G6PD* mutant tumors did not have lower levels of any TCA cycle intermediate as compared to control cells (*SI Appendix*, Fig. S4 *B* and *C*). Loss of *G6PD* function thus reduces the levels of TCA cycle intermediates in some melanomas.

To further explore this, we tested if glutaminase inhibition depleted TCA cycle intermediates to a greater extent in the *G6PD* mutant as compared to control melanomas. CB-839 treatment significantly reduced the levels of at least some TCA cycle intermediates in all three melanomas, particularly in *G6PD* mutant melanomas (*SI Appendix*, Fig. S4 *D*–*F*). In M214 and M481 melanomas, we observed severe depletion of several TCA cycle intermediates upon CB-839 treatment (*SI Appendix*, Fig. S4 *E* and *F*). These data are consistent with the recent study (27) in suggesting that glutamine consumption may be increased in *G6PD* mutant melanomas partly to restore the levels of TCA cycle intermediates that are consumed by increased malic enzyme and isocitrate dehydrogenase function.

## Discussion

Our data suggest that melanoma cells become more dependent upon the oxidative pentose phosphate pathway during metastasis. Melanoma cells experience a spike in ROS during metastasis, leading to oxidative stress and the death of most metastasizing cells by ferroptosis (15, 16, 21, 41). This is observed in both patient-derived xenografts growing in immunocompromised mice, as well as mouse melanomas growing in syngeneic, immunocompetent mice. Oxidative stress limits the metastasis of other cancers as well (42–51), though oxidative stress may promote metastasis under certain circumstances (52, 53). Large clinical trials found that antioxidant supplementation in humans increases cancer incidence and mortality (54–58). Pro-oxidant therapies have the potential to inhibit cancer progression by exacerbating oxidative stress in metastasizing cells and nascent metastatic nodules (17).

To develop effective pro-oxidant therapies, it will be necessary to identify the diverse mechanisms cancer cells use to manage oxidative stress during metastasis. Our results show that melanoma cells have redundant and layered protection against oxidative stress: reduced G6PD function led to compensatory up-regulation of glutaminolysis and malic enzyme activity. This is consistent with results in other cancers, which exhibited compensatory increases in isocitrate dehydrogenase activity, malic enzyme activity, and folate metabolism in response to reduced G6PD function (1, 26, 27). Consequently, reduced G6PD function decreases, but does not completely eliminate, metastasis. The increased glutaminolysis in *G6PD* mutant melanomas decreased oxidative stress and replenished TCA cycle intermediates.

## Materials and Methods

### Melanoma Specimens and Enzymatic Dissociation.

Melanoma specimens were obtained with informed consent from patients according to protocols approved by the Institutional Review Boards (IRB) of the University of Michigan Medical School (IRBMED approvals HUM00050754 and HUM0005008524) and the University of Texas Southwestern Medical Center (IRB approval 102010-051), and were de-identified prior to use in this study. Materials used in this study are available, either commercially or from the authors, though there are restrictions imposed by IRB requirements and institutional policy on the sharing of materials from patients. Single-cell suspensions were obtained by dissociating tumors in Kontes tubes with disposable pestles (VWR) followed by enzymatic dissociation in 200 U/mL collagenase IV (Worthington), DNase (50 U/mL), and 5 mM CaCl_2_ at 37 °C for 20 min. We typically used 1 to 5 mL of dissociation medium, depending on the size of the tumors. After dissociation, cell suspensions were filtered through a 40-μm cell strainer to remove clumps.

### Mouse Studies and Xenograft Assays.

All mouse experiments complied with all relevant ethical regulations and were performed according to protocols approved by the Institutional Animal Care and Use Committee at the University of Texas Southwestern Medical Center (protocol 2016-101360). Melanoma cell suspensions were prepared for injection in staining medium (L15 medium containing bovine serum albumin [1 mg/mL], 1% penicillin/streptomycin, and 10 mM Hepes (pH 7.4) with 25% high-protein Matrigel [product 354248; BD Biosciences]). Subcutaneous injections were performed in the right flank of NOD.CB17-*Prkdc^scid^ Il2rg^tm1Wjl^*/SzJ (NSG) mice in a final volume of 50 μL. Four- to 8-wk-old male and female NSG mice were transplanted with 100 melanoma cells, subcutaneously unless otherwise specified. Both male and female mice were used. Subcutaneous tumor diameters were measured weekly with calipers until any tumor in the mouse cohort reached 2.5 cm in its largest diameter, in agreement with the approved animal protocol. At that point, all mice in the cohort were euthanized and spontaneous metastasis was evaluated by gross inspection of visceral organs for macrometastases and bioluminescence imaging of visceral organs to quantify metastatic disease burden (see details below).

### Bioluminescence Imaging.

All melanomas were tagged with stable luciferase expression, enabling the quantitation of metastatic disease burden by bioluminescence imaging. Five minutes before imaging, mice were injected intraperitoneally with 100 μL of PBS containing D-luciferin monopotassium salt (40 mg/mL) (Goldbio). Mice were anesthetized with isoflurane 2 min before imaging. All mice were imaged using an IVIS Lumina S5 (Perkin-Elmer) with Living Image software (Perkin-Elmer). After completion of whole-body imaging, mice were euthanized and individual organs (including the heart, lung, liver, pancreas, spleen, and kidney) were surgically removed and imaged. The exposure time ranged from 15 to 30 s, depending on the maximum signal intensity, to avoid saturation of the luminescence signal. To measure the background luminescence, a negative control mouse not transplanted with melanoma cells was imaged. The bioluminescence signal (total photon flux) was quantified with “region of interest” measurement tools in Living Image software. Metastatic disease burden was calculated as observed total photon flux from all organs in xenografted mice minus background total photon flux in negative control mice. Negative values were set to 1 for purposes of presentation and statistical analysis.

### Cell Labeling and Flow Cytometry.

Melanoma cells were identified and sorted by flow cytometry as described previously (15, 20). All antibody staining was performed for 20 min on ice, followed by washing with HBSS and centrifugation at 200 × *g* for 5 min. Cells were stained with directly conjugated antibodies against mouse CD45 (APC, eBiosciences), mouse CD31 (APC, Biolegend), mouse Ter119 (APC, eBiosciences), and human HLA-ABC (G46-2.6-FITC, BD Biosciences). Human melanoma cells were isolated as cells that were positive for HLA and negative for mouse endothelial and hematopoietic markers. Cells were washed with staining medium and resuspended in DAPI (1 μg/mL; Sigma) to eliminate dead cells from sorts and analyses. Cells were examined on an LSRFortessa cell analyzer (Becton Dickinson) or sorted on a FACS Fusion Cell Sorter (Becton Dickinson). For analysis of circulating melanoma cells, blood was collected from mice by cardiac puncture with a syringe pretreated with citrate-dextrose solution (Sigma) when subcutaneous tumors reached 2.5 cm in diameter. Red blood cells were sedimented using Ficoll (Ficoll Paque Plus, GE Healthcare). The remaining cells were washed with HBSS (Invitrogen) before antibody staining and flow cytometry.

### CRISPR Editing of *G6PD* in Melanoma Cells.

Single-guide RNAs (sgRNAs) targeting exon 6 of human *G6PD* were designed using publicly available tools (https://zlab.bio/guide-design-resources): *G6PD* sgRNA #1, 5′-CTGGTCCTCACGGAACAGGG-3′; *G6PD* sgRNA #2, 5′-AGGCTGGAACCGCATCATCG-3′. The sgRNAs were cloned into the U6-driven Cas9 expression vector (pX458-pSpCas9(BB)-2AGFP; 48318, Addgene) (59). sgRNA insertion was confirmed by Sanger sequencing.

Approximately 100,000 to 500,000 melanoma cells were plated in tissue-culture–treated six-well plates in DMEM plus 10% FBS and 1% penicillin/streptomycin. One microgram of each of the two sgRNA constructs was cotransfected into the melanoma cells using polyjet (SignaGen) according to the manufacturer’s instructions. After 36 to 48 h, GFP^+^ cells were flow cytometrically isolated and allowed to recover for 24 to 48 h in DMEM plus 10% FBS and 1% penicillin/streptomycin. Single cells were then plated in tissue-culture–treated 96-well plates in Prime-XV tumorsphere medium (Irvine Scientific) supplemented with 2 U/mL heparin (Sigma), 0.1 μg/mL hydrocortisone (Sigma), 2% B27 (Thermo Fisher), 1 μM StemRegenin 1 (StemCell Technologies), 10% charcoal stripped FBS (Thermo Fisher), 10 μg/mL bovine pituitary extract (BPE; Lonza), 10 ng/mL recombinant human interleukin (IL)-8 (CXCL8; Peprotech), 20 ng/mL recombinant human GRO-α/MGSA (CXCL1; Peprotech), and 25 ng/mL recombinant human HGF (Peprotech). Genomic DNA was isolated from individual clones with QuickExtract (Epicenter) and clones were screened and sequenced for *G6PD* deletions.

### G6PD Activity Assay.

G6PD enzyme activity was determined using the G6PD Activity Assay Kit (12581S, Cell Signaling Technology) according to the manufacturer’s instructions. Lysates were collected from subcutaneous melanomas or melanoma cells growing in culture and added to assay buffer. G6P, in the presence of NADP, was oxidized by G6PD to generate 6PGL and NADPH. The NADPH was then amplified by the diaphorase-cycling system to produce fluorescent resorufin molecules. The relative fluorescent units (RFU) was determined using a plate reader with excitation at 540 nm and emission at 590 nm. RFU is proportional to G6PD activity in this assay.

### Metabolomic Analysis.

HILIC chromatographic separation of metabolites was achieved using a Millipore ZIC-pHILIC column (5 μm, 2.1 × 150 mm) with a binary solvent system of 10 mM ammonium acetate in water, pH 9.8 (solvent A) and acetonitrile (solvent B) with a constant flow rate of 0.25 mL/min. For gradient separation, the column was equilibrated with 90% solvent B. After injection, the gradient proceeded as follows: 0 to 15 min linear ramp from 90% B to 30% B; 15 to 18 min isocratic flow of 30% B; 18 to 19 min linear ramp from 30% B to 90% B; 19 to 27 column regeneration with isocratic flow of 90% B. Metabolites were measured with a Thermo Scientific QExactive HF-X hybrid quadrupole orbitrap high-resolution mass spectrometer (HRMS) coupled to a Vanquish UHPLC. HRMS data were acquired with two separate acquisition methods. Individual samples were acquired with an HRMS full scan (precursor ion only) method switching between positive and negative polarities. For data-dependent, high-resolution tandem MS (ddHRMS/MS) methods, precursor ion scans were acquired at a resolving power of 60,000 full width at half-maximum (FWHM) with a mass range of 80 to 1,200 Da. The AGC target value was set to 1 × 10^6^ with a maximum injection time of 100 ms. Pooled samples were generated from an equal mixture of all individual samples and analyzed using individual positive- and negative-polarity spectrometry ddHRMS/MS acquisition methods for high-confidence metabolite identification. Product ion spectra were acquired at a resolving power of 15,000 FWHM without a fixed mass range. The AGC target value was set to 2 × 10^5^ with a maximum injection time of 150 ms. Data-dependent parameters were set to acquire the top 10 ions with a dynamic exclusion of 30 s and a mass tolerance of 5 ppm. Isotope exclusion was turned on and a normalized collision energy of 30 was applied. Settings remained the same in both polarities.

Metabolite identities were confirmed in three ways: 1) precursor ion *m/z* was matched within 5 ppm of theoretical mass predicted by the chemical formula; 2) fragment ion spectra were matched within a 5-ppm tolerance to known metabolite fragments; and 3) the retention time of metabolites was within 5% of the retention time of a purified standard run with the same chromatographic method. Metabolites were relatively quantitated by integrating the chromatographic peak area of the precursor ion searched within a 5-ppm tolerance.

To analyze low-abundance metabolites extracted from tumors (such as NADPH, NADP^+^, NAD^+^, NADH, 6PG, and 6PGL), we created an additional targeted selected ion monitoring (tSIM) scan. We used a resolving power of 60,000 FWHM and an AGC target of 1 × 10^5^ with a maximum injection time of 100 ms for these scan events. An inclusion list for each *m/z* of interest was created with an isolation window of 5 Da and an isolation offset of 1 Da. NADPH/NADP^+^ ratios were determined by integrating the extracted ion chromatograms for NADPH in the negative tSIM scan (*m/z* = 744.0838) and NADP^+^ in the positive tSIM scan (*m*/*z* = 744.0827). Fragmentation spectra from pooled samples were used for structural confirmation of NADPH and NADP^+^.

For analysis of the GSH-to-GSSG ratio by liquid chromatography (LC) MS/MS, subcutaneous tumor fragments weighing 5 to 15 mg were homogenized using Kontes tubes with disposable pestles (VWR) in ice-cold 80:20 methanol:water (vol/vol), with 0.1% formic acid to prevent spontaneous oxidation (60), followed by three freeze–thaw cycles in liquid nitrogen. The supernatant was collected after a 10-min centrifugation at 13,000 × *g* at 4 °C then lyophilized. Lyophilized samples were reconstituted in 100 μL of 0.1% formic acid in water, vortexed, and analyzed by LC-MS/MS. GSH/GSSG analysis was performed using a SCIEX 6500+ Q-Trap mass spectrometer coupled to a Shimadzu LC-20A UHPLC system. Chromatographic separation was carried out with a Waters HSS T3 column and a binary solvent gradient of water with 0.1% formic acid (solvent A) and acetonitrile with 0.1% formic acid (solvent B). The following gradient was used for separation: 0 to 3 min, isocratic flow of 0% B; 3 to 8 min, 0 to 100% B; 8 to 13 min, isocratic flow of 100% B; 13 to 13.1 min, 100 to 0% B; 13.1 to 18 min, isocratic flow of 0% B. The flow rate was held constant at 0.2 mL/min. The mass spectrometer was operated in MRM mode, monitoring the following transitions for GSH, GSSH, and their respective internal standards in positive mode: GSH 308/162; GSSG 613/355; GSH internal standard (ISTD) 311/165; GSSG ISTD 619/165. Transitions and source parameters were optimized by infusion before analysis. GSH/GSSG ratios were calculated based on the molar values of GSH and GSSG, determined using a standard curve and internal standards.

### Isotope Tracing.

We extracted and acquired mass spectra of metabolites from isotopically labeled specimens using the same methods described above. Analysis of fractional enrichment was achieved by calculating the theoretical mass of all ^13^C isotopologues of a metabolite and integrating the resulting peak in the extracted ion chromatogram. We used the following criteria to ensure the correct peaks were integrated for analysis: 1) the precursor ion *m/z* of the M+0 peak was matched within 5 ppm of the theoretical mass predicted by its chemical formula; 2) the retention time of the M+0 peak was within 5% of the retention time of a purified chemical standard run with the same chromatographic method; 3) all isotopes of a potentially labeled metabolite were within 5 ppm of their predicted *m/z* by chemical formula; and 4) all isotopologues eluted simultaneously with the M+0 peak. An unlabeled sample was run alongside all isotopically labeled samples to acquire product ion spectra for additional verification. After analyzing the raw data, peak areas were further analyzed using a published algorithm to correct for naturally abundant isotopes and calculate fractional enrichment (61).

To trace isotopically labeled glucose or glutamine in culture, melanoma cells were grown adherently in six-well plates with DMEM lacking glucose, glutamine, and phenol red (A14430, Gibco), supplemented with 10% dialyzed fetal calf serum, 12.5 mM glucose, and penicillin/streptomycin. At time 0, the cells were washed with PBS and fed 1 mL of culture medium supplemented with 2 mM [U-^13^C]glutamine (CLM-1822, Cambridge Isotope Laboratories) or 12.5 mM [1,2-^13^C]glucose (CLM-504-1, Cambridge Isotope Laboratories). Lysates were harvested in 80% methanol at the time points indicated in the figures and analyzed by LC-MS/MS.

In vivo isotope tracing experiments were performed when subcutaneous tumors reached 1.5 to 2 cm in diameter. Before infusions, mice were fasted for 16 h, then a 27-gauge catheter was placed in the lateral tail vein under anesthesia. We intravenously infused [U-^13^C]glutamine (CLM-1822, Cambridge Isotope Laboratories) as a bolus of 0.1725 mg/g body mass over 1 min in 150 μL of saline, followed by continuous infusion of 0.00288 mg/g body mass/min for 5 h in a volume of 150 μL/h (37). For infusions of [1,2-^13^C]glucose (CLM-504, Cambridge Isotope Laboratories), we intravenously infused a bolus of 0.4125 mg/g body mass over 1 min in 125 μL of saline, followed by continuous infusion of 0.008 mg/g body mass/min for 3 h in a volume of 150 μL/h (36). At the end of the infusion, mice were euthanized and tumors were collected and immediately frozen in liquid nitrogen. To assess the fractional enrichments in plasma, 20 μL of blood was obtained after 30, 60, 120, and 180 min of infusion (glucose) or hourly for 5 h after infusion (glutamine).

### Glutaminase Inhibitor Treatment.

To assess the growth of melanoma cells in the presence of the glutaminase inhibitor, CB-839 (Telaglenastat, S7655, Selleck Chemicals), melanoma cells were grown adherently in 24- or 96-well plates. To test the effects of glutaminase inhibition, melanoma cells were cultured in DMEM + 10% FBS and at day 0 treated with 100 nM CB-839 or DMSO control. To assess the ability of antioxidants to rescue the effects of CB-839, cells were cotreated with glutathione ethyl ester, a cell-permeable derivative of GSH that undergoes hydrolysis by intracellular esterases to release GSH (14953, Cayman) or Trolox [(±)-6-Hydroxy-2,5,7,8-tetramethylchromane-2-carboxylic acid, 238813, Sigma]. Cells were trypsinized at the indicated time points and the number of cells was counted manually using a hemocytometer or using an LSRFortessa flow cytometer. Dead cells were identified as trypan blue or DAPI^+^ and were excluded from the counts.

### Glutamine Uptake and GDH Activity.

Glutamine concentrations in the culture medium were measured using an automated chemical analyzer (NOVA Biomedical). To determine the rate of glutamine consumption by cells, we measured the concentration of glutamine in the culture medium after the cells had been growing for 8 to 24 h and compared it to the concentration of glutamine in culture medium incubated for the same period of time in wells without cells.

GDH activity in cultured melanoma cells was quantitated by measuring the transfer of ^15^N from [α-^15^N]glutamine (NLM-1016-1, Cambridge Isotope Laboratories) to ^15^NH_4_^+^ as described previously (38) after culture for 8 h in the presence of [α-^15^N]glutamine. Total NH_4_^+^ secreted into the medium was measured by spectrophotometry (Megazyme). The fractional enrichment of ^15^N in NH_4_^+^ in the culture medium was determined using a modification of a published method (62): the NH_4_^+^ in the culture medium was conjugated to a ketoacid (ketovaleric acid) in the presence of purified GDH and NADH for 30 min at 37 °C to form an amino acid, α-aminobutyrate. The α-aminobutyrate was then derivatized by addition of trimethylsilyl groups and the samples were purified by applying to an ion exchange column (AG 50W-X4, Bio-Rad), washed with water, and eluted with 2 mL of 4 N ammonium hydroxide. Standards containing known ratios of unlabeled/^15^*N*-labeled NH_4_^+^ were prepared using the same method. All samples were evaporated and derivatized in 100 μL of Tri-Sil (Thermo Scientific), then analyzed by GC/MS with an Agilent 6890N GC coupled to an Agilent 5973 Mass Selective Detector. The oven temperature was ramped from 70 °C to 150 °C by 5 °C/min, then by 10 °C/min to 325 °C. We measured the ratio of aminobutyrate *m/z* 130 (unenriched) to 131 (enriched), corresponding to the unlabeled/^15^*N*-labeled NH_4_^+^ ratio, and then multiplied by the total nMoles of NH_4_^+^ in the medium to determine the molar amount of NH_4_^+^ produced by the cells via GDH.

### Analysis of ROS Levels by Flow Cytometry.

Subcutaneous tumors were surgically excised as quickly as possible after euthanizing the mice and then melanoma cells were mechanically dissociated in 700 μL of staining medium. Single-cell suspensions were obtained by passing the dissociated cells through a 40-μm cell strainer. Equal numbers of dissociated cells from each tumor were stained for cell surface markers and then stained for 30 min at 37 °C with 5 mM CellROX green or CellROX deep red (Life Technologies) in HBSS-free (Ca^2+^ and Mg^2+^-free) with DAPI to distinguish live from dead cells. The cells were then washed and analyzed by flow cytometry using either a FACS Fusion or a LSRFortessa (BD Biosciences) to assess ROS levels in live human melanoma cells (positive for human HLA and dsRed and negative for DAPI and mouse CD45/CD31/Ter119).

### Western Blot Analysis.

Melanomas were excised and quickly snap-frozen in liquid nitrogen. Tumor lysates were prepared in Kontes tubes with disposable pestles using RIPA Buffer (Cell Signaling Technology) supplemented with phenylmethylsulphonyl fluoride (Sigma), and protease and phosphatase inhibitor mixture (Roche). The bicinchoninic acid protein assay (Thermo) was used to quantify protein concentrations. Equal amounts of protein (5 to 20 μg) were loaded into each lane and separated on 4 to 20% polyacrylamide Tris glycine SDS gels (Bio-Rad), then transferred to polyvinylideneifluoride membranes (Bio-Rad). The membranes were blocked for 1 h at room temperature with 5% milk or 5% BSA in TBS supplemented with 0.1% Tween-20 (TBST) and then incubated with primary antibodies overnight at 4 °C. After washing, then incubating with horseradish peroxidase-conjugated secondary antibody (Cell Signaling Technology), signals were developed using SuperSignal West (Thermo Fisher). Blots were sometimes stripped using Restore stripping buffer (Thermo Fisher) and restained with other primary antibodies. The following antibodies were used for Western blots: anti-G6PD (D5D2, Cell Signaling Technologies), anti-ME1 (PA5-21550, Thermo Scientific), anti-ME2 (12399, Cell Signaling Technologies), anti-GAPDH (14C10, Cell Signaling Technologies), and anti–β-actin (D6A8, Cell Signaling Technologies).

### Statistical Methods.

Figures generally reflect data obtained in multiple independent experiments performed using different mice or cultures on different days. Mice were allocated to experiments randomly and samples processed in an arbitrary order, but formal randomization techniques were not used. Before analyzing the statistical significance of differences among treatments, we tested whether data were normally distributed and whether variance was similar among treatments. To test for normality, we performed the Shapiro–Wilk tests when 3 ≤ *n* < 20 or D’Agostino omnibus tests when *n* ≥ 20. To test whether variability significantly differed among treatments we performed F-tests (for experiments with two treatments) or Levene’s median tests (for experiments with more than two treatments). When the data significantly deviated from normality or variability significantly differed among treatments, we log_2_-transformed the data and tested again for normality and variability. If the transformed data no longer significantly deviated from normality and equal variability, we performed parametric tests on the transformed data. If log_2_-transformation was not possible or the transformed data still significantly deviated from normality or equal variability, we performed nonparametric tests on the nontransformed data.

All of the statistical tests we used were two-sided. To assess the statistical significance of a difference between two treatments, we used Student’s *t* tests (when a parametric test was appropriate), Welch’s *t* tests (when data were normally distributed but not equally variable), or Mann–Whitney *U* tests (when a nonparametric test was appropriate). Multiple *t* tests (parametric or nonparametric) were followed by the false-discovery rate (FDR) multiple comparisons adjustment. To assess the statistical significance of differences in median fluorescence intensity of CellRox green staining, we used a Welch’s one-way ANOVA (when data were normally distributed but unequally variable) followed by the Dunnett’s T3 method for multiple comparisons adjustment. To assess the statistical significance of differences in metabolite levels between subcutaneous and metastatic tumors, we used repeated-measures two-way ANOVAs (samples were matched, and a parametric test was appropriate). To assess the statistical significance of melanoma cell growth over time in culture, we used the nparLD method (63), a statistical tool for nonparametric longitudinal data analysis. To assess the statistical significance of differences between multiple control clones and multiple *G6PD^mut^* clones, we performed linear mixed-effects analysis or generalized linear mixed-effects analysis by combining data from A375, M214, and M481. Multiple comparisons were adjusted using the FDR method. All statistical analyses were performed using Graphpad Prism 9.2.0 or R 4.0.2 with the stats, fBasics, car, lme4, emmeans, and nparLD packages. All data are mean ± SD.

No data were excluded; however, mice sometimes died during experiments, presumably due to the growth of metastatic tumors. In those instances, data that had already been collected on the mice in interim analyses were included (such as subcutaneous tumor growth measurements over time) even if it was not possible to perform the end-point analysis of metastatic disease burden due to the premature death of the mice.

## Supplementary Material

Supplementary File

## Data Availability

All study data are included in the main text and *SI Appendix*.

## References

[1] L. Chen , NADPH production by the oxidative pentose-phosphate pathway supports folate metabolism. Nat. Metab. 1, 404–415 (2019). 31058257 PMC6489125

[2] P. P. Pandolfi , Targeted disruption of the housekeeping gene encoding glucose 6-phosphate dehydrogenase (G6PD): G6PD is dispensable for pentose synthesis but essential for defense against oxidative stress. EMBO J. 14, 5209–5215 (1995). 7489710 10.1002/j.1460-2075.1995.tb00205.x PMC394630

[3] A. S. Alving, P. E. Carson, C. L. Flanagan, C. E. Ickes, Enzymatic deficiency in primaquine-sensitive erythrocytes. Science 124, 484–485 (1956). 10.1126/science.124.3220.484-a 13360274

[4] A. Kuehne , Acute activation of oxidative pentose phosphate pathway as first-line response to oxidative stress in human skin cells. Mol. Cell 59, 359–371 (2015). 26190262 10.1016/j.molcel.2015.06.017

[5] R. A. Cairns, I. S. Harris, T. W. Mak, Regulation of cancer cell metabolism. Nat. Rev. Cancer 11, 85–95 (2011). 21258394 10.1038/nrc2981

[6] L. Longo , Maternally transmitted severe glucose 6-phosphate dehydrogenase deficiency is an embryonic lethal. EMBO J. 21, 4229–4239 (2002). 12169625 10.1093/emboj/cdf426 PMC126165

[7] C. J. Nicol, J. Zielenski, L. C. Tsui, P. G. Wells, An embryoprotective role for glucose-6-phosphate dehydrogenase in developmental oxidative stress and chemical teratogenesis. FASEB J. 14, 111–127 (2000). 10627286 10.1096/fasebj.14.1.111

[8] E. T. Nkhoma, C. Poole, V. Vannappagari, S. A. Hall, E. Beutler, The global prevalence of glucose-6-phosphate dehydrogenase deficiency: A systematic review and meta-analysis. Blood Cells Mol. Dis. 42, 267–278 (2009). 19233695 10.1016/j.bcmd.2008.12.005

[9] T. Vulliamy, P. Mason, L. Luzzatto, The molecular basis of glucose-6-phosphate dehydrogenase deficiency. Trends Genet. 8, 138–143 (1992). 1631957 10.1016/0168-9525(92)90372-B

[10] E. Beutler, Glucose-6-phosphate dehydrogenase deficiency. N. Engl. J. Med. 324, 169–174 (1991). 1984194 10.1056/NEJM199101173240306

[11] P. Cocco, Does G6PD deficiency protect against cancer? A critical review. J. Epidemiol. Community Health 41, 89–93 (1987). 3309118 10.1136/jech.41.2.89 PMC1052590

[12] G. M. Pes, G. Bassotti, M. P. Dore, Colorectal cancer mortality in relation to glucose-6-phosphate dehydrogenase deficiency and consanguinity in sardinia: A spatial correlation analysis. Asian Pac. J. Cancer Prev. 18, 2403–2407 (2017). 28950694 10.22034/APJCP.2017.18.9.2403 PMC5720643

[13] G. M. Pes, A. Errigo, S. Soro, N. P. Longo, M. P. Dore, Glucose-6-phosphate dehydrogenase deficiency reduces susceptibility to cancer of endodermal origin. Acta Oncol. 58, 1205–1211 (2019). 31109224 10.1080/0284186X.2019.1616815

[14] M. P. Dore, A. Davoli, N. Longo, G. Marras, G. M. Pes, Glucose-6-phosphate dehydrogenase deficiency and risk of colorectal cancer in Northern Sardinia: A retrospective observational study. Medicine (Baltimore) 95, e5254 (2016). 27858887 10.1097/MD.0000000000005254 PMC5591135

[15] E. Piskounova , Oxidative stress inhibits distant metastasis by human melanoma cells. Nature 527, 186–191 (2015). 26466563 10.1038/nature15726 PMC4644103

[16] K. Le Gal , Antioxidants can increase melanoma metastasis in mice. Sci. Transl. Med. 7, 308re8 (2015). 10.1126/scitranslmed.aad3740 26446958

[17] A. Tasdogan, J. M. Ubellacker, S. J. Morrison, Redox regulation in cancer cells during metastasis. Cancer Discov. 11, 2682–2692 (2021). 34649956 10.1158/2159-8290.CD-21-0558 PMC8563381

[18] I. S. Harris , Glutathione and thioredoxin antioxidant pathways synergize to drive cancer initiation and progression. Cancer Cell 27, 211–222 (2015). 25620030 10.1016/j.ccell.2014.11.019

[19] Z. T. Schafer , Antioxidant and oncogene rescue of metabolic defects caused by loss of matrix attachment. Nature 461, 109–113 (2009). 19693011 10.1038/nature08268 PMC2931797

[20] A. Tasdogan , Metabolic heterogeneity confers differences in melanoma metastatic potential. Nature 577, 115–120 (2020). 31853067 10.1038/s41586-019-1847-2 PMC6930341

[21] J. M. Ubellacker , Lymph protects metastasizing melanoma cells from ferroptosis. Nature 585, 113–118 (2020). 32814895 10.1038/s41586-020-2623-z PMC7484468

[22] T. Hu , Variant G6PD levels promote tumor cell proliferation or apoptosis via the STAT3/5 pathway in the human melanoma xenograft mouse model. BMC Cancer 13, 251 (2013). 23693134 10.1186/1471-2407-13-251 PMC3765728

[23] R. Lin , 6-Phosphogluconate dehydrogenase links oxidative PPP, lipogenesis and tumour growth by inhibiting LKB1-AMPK signalling. Nat. Cell Biol. 17, 1484–1496 (2015). 26479318 10.1038/ncb3255 PMC4628560

[24] C. Shan , Lysine acetylation activates 6-phosphogluconate dehydrogenase to promote tumor growth. Mol. Cell 55, 552–565 (2014). 25042803 10.1016/j.molcel.2014.06.020 PMC4142084

[25] R. Liu , Tyrosine phosphorylation activates 6-phosphogluconate dehydrogenase and promotes tumor growth and radiation resistance. Nat. Commun. 10, 991 (2019). 30824700 10.1038/s41467-019-08921-8 PMC6397164

[26] J. M. Ghergurovich , Glucose-6-phosphate dehydrogenase is not essential for K-Ras-driven tumor growth or metastasis. Cancer Res. 80, 3820–3829 (2020). 32661137 10.1158/0008-5472.CAN-19-2486 PMC7501231

[27] H. Ding , Activation of the NRF2 antioxidant program sensitizes tumors to G6PD inhibition. Sci. Adv. 7, eabk1023 (2021). 34788087 10.1126/sciadv.abk1023 PMC8598006

[28] E. I. Chen , Adaptation of energy metabolism in breast cancer brain metastases. Cancer Res. 67, 1472–1486 (2007). 17308085 10.1158/0008-5472.CAN-06-3137

[29] O. G. McDonald , Epigenomic reprogramming during pancreatic cancer progression links anabolic glucose metabolism to distant metastasis. Nat. Genet. 49, 367–376 (2017). 28092686 10.1038/ng.3753 PMC5695682

[30] N. Deshpande, I. Mitchell, R. Millis, Enzyme studies in human breast tumours. Eur. J. Cancer 13, 1261–1267 (1977). 145367 10.1016/0014-2964(77)90034-2

[31] H. Pu , Overexpression of G6PD is associated with high risks of recurrent metastasis and poor progression-free survival in primary breast carcinoma. World J. Surg. Oncol. 13, 323 (2015). 26607846 10.1186/s12957-015-0733-0 PMC4660828

[32] J. Wang , Overexpression of G6PD is associated with poor clinical outcome in gastric cancer. Tumour Biol. 33, 95–101 (2012). 22012600 10.1007/s13277-011-0251-9

[33] H. Q. Ju , Disrupting G6PD-mediated redox homeostasis enhances chemosensitivity in colorectal cancer. Oncogene 36, 6282–6292 (2017). 28692052 10.1038/onc.2017.227 PMC5684443

[34] S. Gómez-Manzo , Glucose-6-phosphate dehydrogenase: Update and Analysis of new mutations around the world. Int. J. Mol. Sci. 17, 2069 (2016). 10.3390/ijms17122069 PMC5187869 27941691

[35] C. Jang, L. Chen, J. D. Rabinowitz, Metabolomics and isotope tracing. Cell 173, 822–837 (2018). 29727671 10.1016/j.cell.2018.03.055 PMC6034115

[36] B. Faubert , Lactate metabolism in human lung tumors. Cell 171, 358–371.e9 (2017). 28985563 10.1016/j.cell.2017.09.019 PMC5684706

[37] I. Marin-Valencia , Analysis of tumor metabolism reveals mitochondrial glucose oxidation in genetically diverse human glioblastomas in the mouse brain in vivo. Cell Metab. 15, 827–837 (2012). 22682223 10.1016/j.cmet.2012.05.001 PMC3372870

[38] C. Yang , Glioblastoma cells require glutamate dehydrogenase to survive impairments of glucose metabolism or Akt signaling. Cancer Res. 69, 7986–7993 (2009). 19826036 10.1158/0008-5472.CAN-09-2266 PMC2764330

[39] L. Li , Discovery and development of small molecule modulators targeting glutamine metabolism. Eur. J. Med. Chem. 163, 215–242 (2019). 30522056 10.1016/j.ejmech.2018.11.066

[40] M. I. Gross , Antitumor activity of the glutaminase inhibitor CB-839 in triple-negative breast cancer. Mol. Cancer Ther. 13, 890–901 (2014). 24523301 10.1158/1535-7163.MCT-13-0870

[41] X. Hong , The lipogenic regulator SREBP2 induces transferrin in circulating melanoma cells and suppresses ferroptosis. Cancer Discov. 11, 678–695 (2021). 33203734 10.1158/2159-8290.CD-19-1500 PMC7933049

[42] F. Dupuy , PDK1-dependent metabolic reprogramming dictates metastatic potential in breast cancer. Cell Metab. 22, 577–589 (2015). 26365179 10.1016/j.cmet.2015.08.007

[43] D. Samanta , PHGDH expression is required for mitochondrial redox homeostasis, breast cancer stem cell maintenance, and lung metastasis. Cancer Res. 76, 4430–4442 (2016). 27280394 10.1158/0008-5472.CAN-16-0530

[44] M. Rios Garcia , Acetyl-CoA carboxylase 1-dependent protein acetylation controls breast cancer metastasis and recurrence. Cell Metab. 26, 842–855.e5 (2017). 29056512 10.1016/j.cmet.2017.09.018

[45] H. Wang , NRF2 activation by antioxidant antidiabetic agents accelerates tumor metastasis. Sci. Transl. Med. 8, 334ra351 (2016). 10.1126/scitranslmed.aad6095 27075625

[46] Z. V. Zou , Antioxidants promote intestinal tumor progression in mice. Antioxidants 10, 241 (2021). 33557356 10.3390/antiox10020241 PMC7915500

[47] L. Lignitto , Nrf2 activation promotes lung cancer metastasis by inhibiting the degradation of Bach1. Cell 178, 316–329.e18 (2019). 31257023 10.1016/j.cell.2019.06.003 PMC6625921

[48] C. Wiel , BACH1 stabilization by antioxidants stimulates lung cancer metastasis. Cell 178, 330–345.e22 (2019). 31257027 10.1016/j.cell.2019.06.005

[49] I. Godet , Fate-mapping post-hypoxic tumor cells reveals a ROS-resistant phenotype that promotes metastasis. Nat. Commun. 10, 4862 (2019). 31649238 10.1038/s41467-019-12412-1 PMC6813355

[50] C. F. Labuschagne, E. C. Cheung, J. Blagih, M. C. Domart, K. H. Vousden, Cell clustering promotes a metabolic switch that supports metastatic colonization. Cell Metab. 30, 720–734.e5 (2019). 31447323 10.1016/j.cmet.2019.07.014 PMC6863392

[51] S. Deghan Manshadi , Folic acid supplementation promotes mammary tumor progression in a rat model. PLoS One 9, e84635 (2014). 24465421 10.1371/journal.pone.0084635 PMC3897399

[52] I. I. C. Chio, D. A. Tuveson, ROS in cancer: The burning question. Trends Mol. Med. 23, 411–429 (2017). 28427863 10.1016/j.molmed.2017.03.004 PMC5462452

[53] E. C. Cheung , Dynamic ROS control by TIGAR regulates the initiation and progression of pancreatic cancer. Cancer Cell 37, 168–182.e4 (2020). 31983610 10.1016/j.ccell.2019.12.012 PMC7008247

[54] S. P. Fortmann, B. U. Burda, C. A. Senger, J. S. Lin, E. P. Whitlock, Vitamin and mineral supplements in the primary prevention of cardiovascular disease and cancer: An updated systematic evidence review for the U.S. Preventive Services Task Force. Ann. Intern. Med. 159, 824–834 (2013). 24217421 10.7326/0003-4819-159-12-201312170-00729

[55] Alpha-Tocopherol, Beta Carotene Cancer Prevention Study Group, The effect of vitamin E and beta carotene on the incidence of lung cancer and other cancers in male smokers. N. Engl. J. Med. 330, 1029–1035 (1994). 8127329 10.1056/NEJM199404143301501

[56] E. A. Klein , Vitamin E and the risk of prostate cancer: The Selenium and Vitamin E Cancer Prevention Trial (SELECT). JAMA 306, 1549–1556 (2011). 21990298 10.1001/jama.2011.1437 PMC4169010

[57] G. E. Goodman , The Beta-Carotene and Retinol Efficacy Trial: Incidence of lung cancer and cardiovascular disease mortality during 6-year follow-up after stopping beta-carotene and retinol supplements. J. Natl. Cancer Inst. 96, 1743–1750 (2004). 15572756 10.1093/jnci/djh320

[58] M. Ebbing , Cancer incidence and mortality after treatment with folic acid and vitamin B12. JAMA 302, 2119–2126 (2009). 19920236 10.1001/jama.2009.1622

[59] F. A. Ran , Genome engineering using the CRISPR-Cas9 system. Nat. Protoc. 8, 2281–2308 (2013). 24157548 10.1038/nprot.2013.143 PMC3969860

[60] B. P. Tu , Cyclic changes in metabolic state during the life of a yeast cell. Proc. Natl. Acad. Sci. U.S.A. 104, 16886–16891 (2007). 17940006 10.1073/pnas.0708365104 PMC2040445

[61] X. Su, W. Lu, J. D. Rabinowitz, Metabolite spectral accuracy on orbitraps. Anal. Chem. 89, 5940–5948 (2017). 28471646 10.1021/acs.analchem.7b00396 PMC5748891

[62] J. T. Brosnan , Alanine metabolism in the perfused rat liver. Studies with (15)N. J. Biol. Chem. 276, 31876–31882 (2001). 11423541 10.1074/jbc.M103890200

[63] K. Noguchi, Y. R. Gel, E. Brunner, F. Konietschke, nparLD: An R software package for the nonparametric analysis of longitudinal data in factorial experiments. J. Stat. Softw. 50, 1–23 (2012).25317082

