# Supplementary material for: Loss of glucose 6-phosphate dehydrogenase function increases oxidative stress and glutaminolysis in metastasizing melanoma cells
Source: Proc Natl Acad Sci U S A. 2022 Feb 2;119(6):e2120617119. doi: 10.1073/pnas.2120617119 (PMC8833200; doi:10.1073/pnas.2120617119)
Supplement: Supplementary File [file pnas.2120617119.sapp.pdf]

PNAS
www.pnas.org

Supplementary Information for

Loss of G6PD function increases oxidative stress and glutaminolysis in metastasizing melanoma cells

Arin B. Aurora[a,1*], Vishal Khivansara[a], Ashley Leach[a], Jennifer G. Gill[a,c], Misty Martin-Sandoval[a], Chendong Yang[a,d], Stacy Y. Kasitinon[a], Divya Bezwada[a], Alpaslan Tasdogan[a], Wen Gu[a,d], Thomas Mathews[a], Zhiyu Zhao[a], Ralph J. DeBerardinis[a,b,d], Sean J. Morrison[a,b,1*]

Sean J. Morrison
Email: sean.morrison@utsouthwestern.edu

Arin B. Aurora
Email: arin.aurora@utsouthwestern.edu

This PDF file includes:
Figures S1 to S4

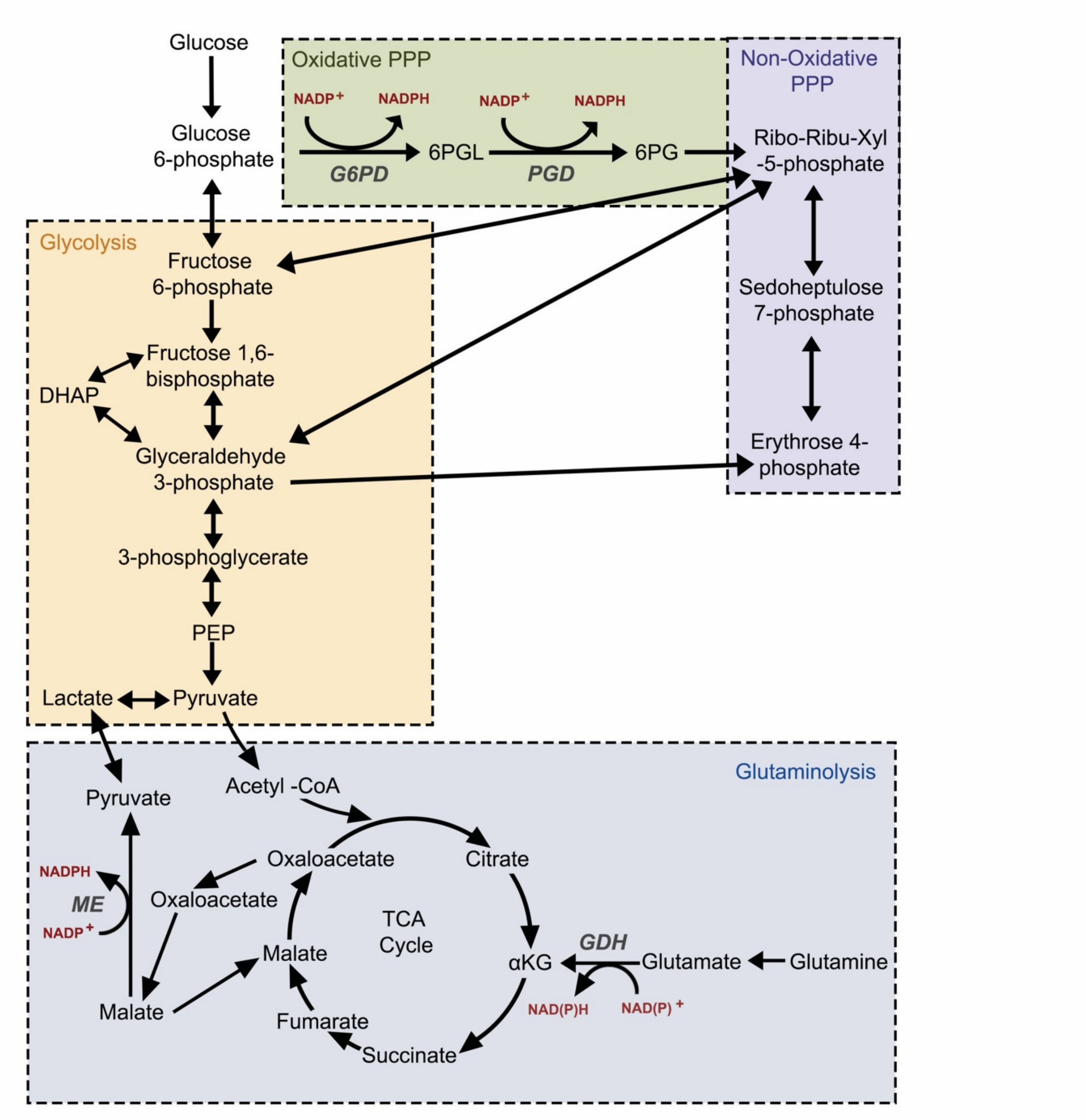

Supplementary Figure 1: A schematic of the oxidative and non-oxidative branches of the pentose phosphate pathway as well as glycolysis and the TCA cycle. The schematic includes all enzymes and metabolites mentioned in our studies. Metabolite abbreviations that are not defined within the figure include: dihydroxyacetone phosphate (DHAP), 6-phosphogluconolactone (6PGL), 6-phosphogluconate (6PG), Ribulose 5-phosphate/Ribose 5-phosphate/Xylulose 5-phosphate (R-R-X-5P), alpha-ketoglutarate (αKG), glutamine (Gln), and glutamate (Glu).

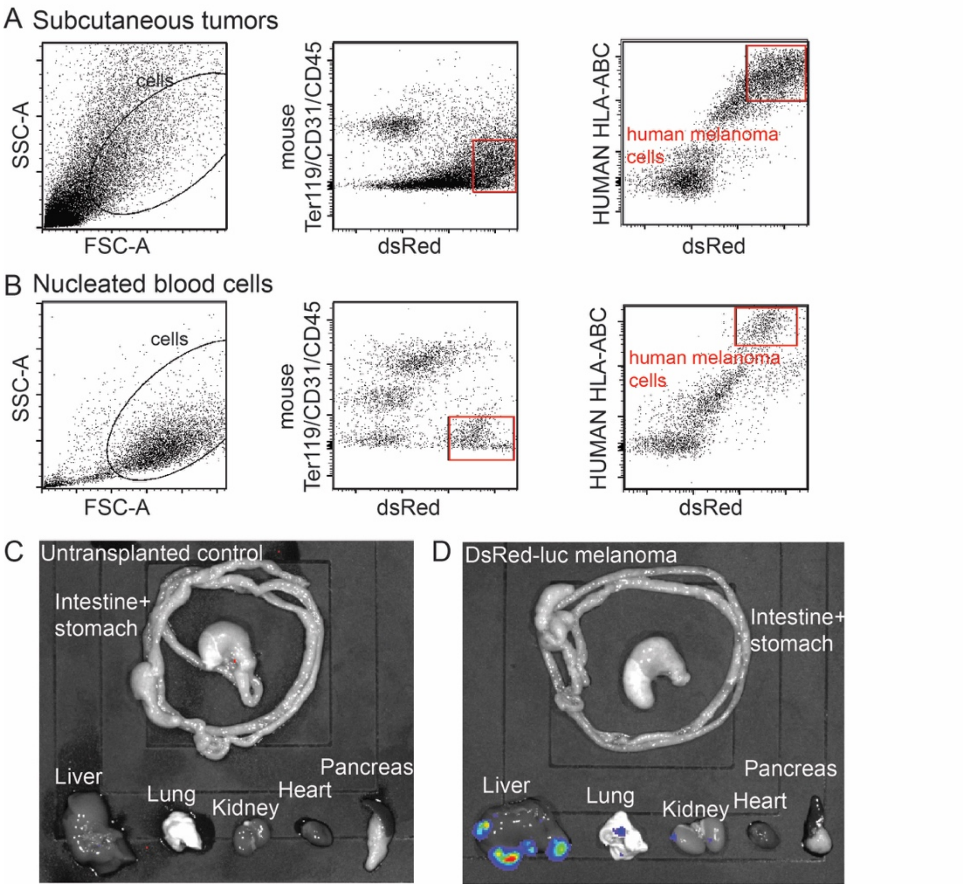

Supplementary Figure 2: The identification of human melanoma cells by flow cytometry and assessment of metastatic disease burden by bioluminescence imaging. (A-B). Flow cytometry plots showing the gating strategies used to identify human melanoma cells in subcutaneous tumors (*A*) or nucleated blood cells (*B*) obtained from xenografted mice. Cells were gated on forward versus side scatter (FSC-A versus SSC-A) to exclude red blood cells, debris, and clusters of cells. Human melanoma cells were selected by including cells that stained positively for DsRed (stably expressed in all melanoma lines) and HLA and excluding cells that stained positively for the mouse hematopoietic and endothelial markers CD45, CD31 or Ter119. (C-D) Visceral organs were surgically removed from each mouse at the end of each experiment and imaged to identify macrometastases and micrometastases and to

determine bioluminescence signal intensity. Each melanoma constitutively expressed luciferase. Shown are representative images from an untransplanted negative control mouse (*C*), illustrating background bioluminescence, and a mouse subcutaneously transplanted with dsRed-luciferase expressing melanoma cells (*D*) with metastases in the liver, lung, and kidney.

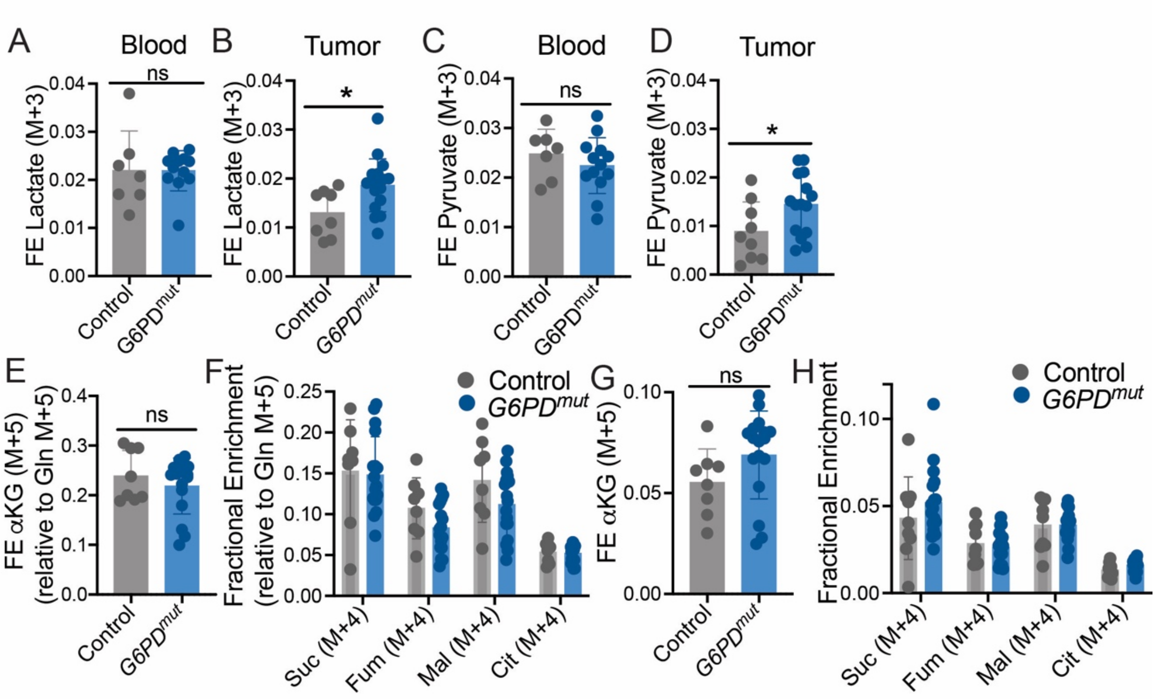

Supplementary Figure 3: Mass isotopomer analysis of blood and tumors from mice infused with [U-^{13}C] glutamine. (A-D) The fractional enrichments of lactate (m+3) (*A, B*) and pyruvate (m+3) (*C, D*) after 5 hours of infusion with [U-^{13}C] glutamine were assessed in the blood (*A,C*) and in primary subcutaneous tumors (*B, D*) of NSG mice xenografted with *G6PD* mutant or control melanomas. (E-H) The fractional enrichments of α-ketoglutarate (m+5) (*E*) or other TCA cycle metabolites (m+4) (*F*) normalized to glutamine (m+5) in control or *G6PD* mutant subcutaneous tumors. The same data without normalization to glutamine (m+5) (*G-H*) (n= 2-3 clones per genotype per melanoma, with 4-8 mice per clone in 2 independent experiments). Each dot represents a different tumor in a different mouse. Statistical significance was assessed using Mann-Whitney tests (A-H). Statistical tests were two-sided. Multiple comparisons were adjusted by the FDR method. *P<0.05, ns=not significant. When no p-value is shown (*F,H*), differences were not statistically significant between control and *G6PD* mutants.

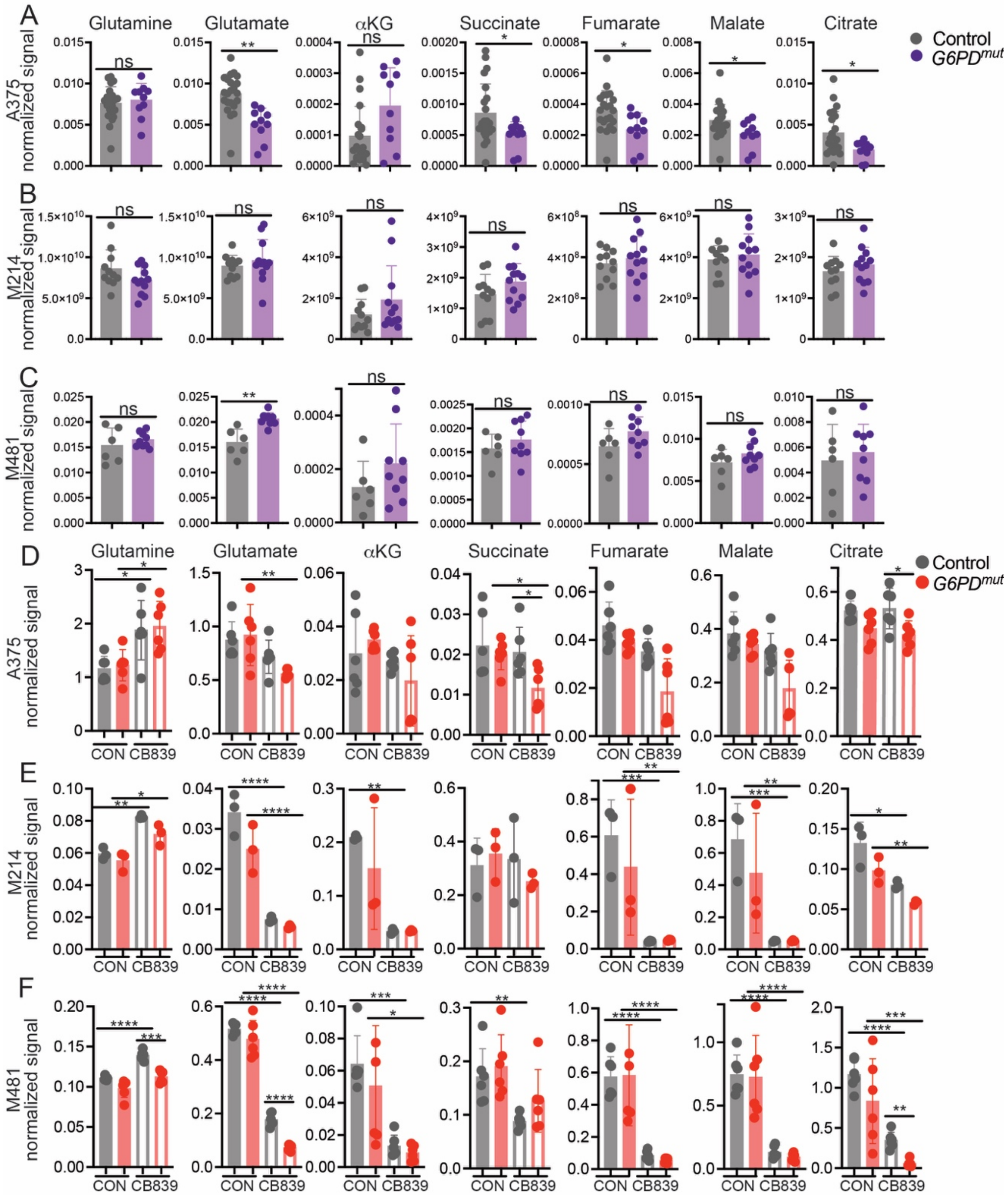

Supplementary Figure 4: The relative levels of TCA cycle intermediates in control and *G6PD* mutant melanomas. (A-C) Metabolomic analysis of TCA cycle intermediates in subcutaneous tumors from *G6PD* mutant and control A375 (*A*), M214 (*B*), and M481 (*C*) melanomas (n=2-3 clones per genotype per melanoma, with 4-8 mice per clone in 2

independent experiments). One representative, of two experiments total, is shown for A-C. Each dot represents a different tumor from a different mouse. (D-F) Metabolomic analysis of TCA cycle intermediates in *G6PD* mutant and control melanomas cultured with or without CB-839 (100nM) for A375 (*D*), M214 (*E*), and M481 (*F*) melanomas (n=2-3 clones per genotype per melanoma, with 3-6 replicates per clone in 1-2 independent experiments per melanoma). Statistical significance was assessed using t-tests, Welch's t-tests or Mann-Whitney tests followed by the FDR method for multiple comparisons adjustment (*A*-*C*) or by using one-way ANOVAs followed by Tukey's method for multiple comparisons adjustment (*D*-*F*) or Welch's one-way ANOVAs followed by Dunnett's T3 method for multiple comparisons adjustment (*D*-*F*). All tests were done with log2-transformed data (D-F). *P<0.05, **P<0.01, ***P<0.001, ****P<0.0001, ns=not significant. Differences are not significant where no p-value is indicated (*D*-*F*).
